# Cytokine profiling reveals increased serum inflammatory cytokines in idiopathic choroidal neovascularization

**DOI:** 10.1186/s12886-019-1101-6

**Published:** 2019-04-24

**Authors:** Shenchao Guo, Houfa Yin, Mingjie Zheng, Yizhen Tang, Bing Lu, Xinyi Chen, Qiuli Fu, Zhenwei Qin, Danni Lyu, Qiaomei Tang, Lifang Zhang, Jian Ma, Li Zhang, Xiaoyun Fang

**Affiliations:** 1 Eye Center, the Second Affiliated Hospital, Zhejiang University School of Medicine, Hangzhou, China; 2 Institute of Translational Medicine, Zhejiang University, Hangzhou, China

**Keywords:** Idiopathic choroidal neovascularization, Serum, Inflammatory cytokines

## Abstract

**Background:**

The exact pathogenesis of idiopathic choroidal neovascularization (ICNV) remains unclear. Cytokine-mediated inflammation has been thought to be involved in the pathophysiology of ICNV. The purpose of this study was to investigate serum cytokine profiles in patients with ICNV and to explore the relationship between serum cytokine levels and ICNV severity.

**Methods:**

This case-control study was conducted in 32 ICNV patients and 30 healthy volunteers. Clinical and demographic information was obtained from the medical data platform and the serum was analysed with a multiplex assay to determine the levels of seven cytokines: interleukin (IL)-2, IL-10, IL-15, IL-17, basic fibroblast growth factor (basic FGF), granulocyte-macrophage colony-stimulating factor (GM-CSF), and vascular endothelial growth factor (VEGF).

**Results:**

Serum levels of IL-2, IL-10, IL-17, basic FGF, and VEGF were elevated in ICNV patients compared to controls. Serum GM-CSF levels were positively related to central retinal thickness, and serum IL-17 levels were positively related to CNV lesion area.

**Conclusion:**

Serum inflammatory cytokines were significantly elevated in ICNV patients compared to controls. This suggests that systemic inflammation may play a critical role in the physiopathology of ICNV.

## Background

Idiopathic choroidal neovascularization (ICNV) is defined as CNV occurring in patients younger than 50 years with no detectable primary ocular or systemic diseases, such as pathologic myopia (PM), angioid streak, trauma, or other inflammatory or hereditary disorders [1, 2]. Although the natural progression and the visual prognosis of ICNV are more favorable than for CNV attributable to age-related macular degeneration (AMD), and majorities of patients with ICNV have good response to anti-vascular endothelial growth factor (VEGF) therapy, some of these patients nonetheless have persistent CNV, and even decreased vision, and need repeated anti-VEGF therapy [3–6].

Although the underlying molecular mechanisms of CNV are not fully understood, accumulated evidence has demonstrated that inflammation plays an important role in the pathogenesis of CNV [7, 8]. Cytokines control inflammation, and have important roles in the pathology of CNV, representing potential targets for clinical intervention [9, 10]. For example, interferon (IFN)-γ, tumor necrosis factor (TNF)-α, and interleukin (IL)-1 are considered to enhance the secretion of VEGF by human retinal pigment epithelial and choroidal fibroblast cells [11]. Over the past decade, the cytokine profile has been extensively studied in the aqueous humour of neovascular AMD patients [12–14]. Systemic levels of inflammatory cytokines in neovascular AMD have also been investigated [15, 16]. It has been suggested that those with more advanced AMD, have higher systemic levels of inflammatory cytokines [15]. However, little is known about the systemic cytokine profile in patients with ICNV. Understanding the cytokine profile, especially inflammatory cytokines, may further elucidate the underlying pathogenic mechanisms of ICNV. Recently, we found significantly increased intraocular levels of IL-2, IL-10, IL-15, IL-17, basic fibroblast growth factor (basic FGF), and granulocyte-macrophage colony-stimulating factor (GM-CSF) in patients with ICNV [17]. In this study, we used a multiplex assay to screen the serum levels of these cytokines and VEGF in patients with ICNV as well as healthy control participants, investigating whether specific cytokines are associated with clinical manifestations.

## Methods

### Study subjects

Thirty-two patients with naive ICNV were recruited from the Eye Center, the Second Affiliated Hospital, Zhejiang University School of Medicine between May 2017 and March 2018. Naive ICNV was determined as newly diagnosed active ICNV without any previous treatment. Inclusion criteria were: newly diagnosed active ICNV undergoing first-time ranibizumab treatment, and age less than 50 years at the time of diagnosis. Diagnosis of ICNV was based on funduscopy, spectral domain optic coherence tomography (SD-OCT, Cirrus OCT; Carl Zeiss Meditec, Dublin, CA, USA) and fundus fluorescein angiography (FFA, Heidelberg Engineering HRA Spectralis, Heidelberg, Germany).

All patients received a comprehensive ophthalmological examination at baseline, including best corrected visual acuity (BCVA) testing, intraocular pressure measurement, dilated fundus examination, OCT and FFA. BCVA was measured by certified operators using a standard Snellen chart. For statistical analysis, BCVA were converted to logarithm of the minimum angle of resolution (logMAR) units. Central retinal thickness (CRT, thickness of the 1-mm central retina) was measured by OCT; lesion area was measured on fluorescein angiograms as described previously [18]. Our exclusion criteria are as follows: (1) clinical features suggesting that CNV was secondary to other causes such as AMD, PM, trauma, choroiditis, or hereditary diseases in the studied or contralateral eye; (2) axial length > 26.0 mm or myopia> − 6 diopter; (3) prior local or systemic treatment for ICNV, or previous intraocular surgery; (4) systemic inflammatory diseases such as autoimmune disease, diabetes mellitus or malignoma.

For comparison, healthy volunteers less than 50 years old (by clinical anamnesis, examination, and blood analysis) with no evidence of active ocular or systemic disease who were undergoing health checkup were recruited as a control group. Blood samples were collected aseptically, and serum was prepared and stored at − 80 °C until cytokine analysis. For patients with ICNV, blood samples were collected before FFA or ranibizumab treatment.

The study protocol was approved by the Ethics Committee of the Second Affiliated Hospital, Zhejiang University School of Medicine, and the research followed the tenets of the Declaration of Helsinki. Written consent was obtained from all patients and controls.

### Cytokine determination in serum samples

The Bio-Plex multiplex assay (Bio-Plex Human Cytokine 7-plex panel; Bio-Rad, Hercules, CA, USA) and the multiplex bead analysis system (Bio-Plex Suspension Array System; Bio-Rad) were used simultaneously to measure serum levels of IL-2, IL-10, IL-15, IL-17, basic FGF, GM-CSF, and VEGF as in a previous study [17]. The levels of serum cytokines were set to 0 if the levels were below detection.

### Statistical analysis

Statistical analyses were performed using SPSS software version 17.0 (SPSS, Inc., Chicago, IL, USA). The χ^2^ test or Fisher exact test was used to compare categorical variables. For continuous variables the unpaired *t* test was used when the data were normally distributed and the Mann-Whitney *U* test when the distribution was skewed. The Spearman’s correlation analysis was used to evaluate the relationship between numerical data. *P* < 0.05 was considered to be statistically significant.

## Results

### Clinical characteristics

In this study, we included 32 ICNV patients and 30 control subjects. The average age of patients with ICNV was 32.9 ± 8.0 years. Seven patients (22%) were male and 25 patients (78%) were female. The average age of control subjects was 33.7 ± 11.5 years. Nine patients (30%) were male and 21 patients (70%) were female. There were no significant differences in mean age (*P* = 0.737) or sex (*P* = 0.465) between the two groups. None of the patients or control was smoking.

In all the 32 patients with ICNV, only one eye of each patient was involved. At baseline, mean BCVA was 0.63 ± 0.37 logMAR, mean CRT was 360.09 ± 76.99 μm, and mean lesion area was 2.46 ± 1.61 mm^2^.

### ICNV and control serum cytokine profiles

Differences in serum cytokine profiles between patients with ICNV and control subjects were analysed and are listed in Table 1. Compared to the control subjects, the levels of IL-2, IL-10, IL-17, basic FGF, and VEGF were significantly elevated in patients with ICNV. IL-15 levels, although not statistically different, tended to be higher in patients with ICNV compared to control subjects. No significant differences were observed in the levels of GM-CSF.

**Table 1:** Cytokine levels in serum of patients with ICNV and control subject

	ICNV Group (*n* = 32)	Control Group (*n* = 30)	*P* Value
IL-2	13.52 ± 5.71	11.38 ± 0.95	< 0.001
IL-10	14.81 ± 8.58	6.60 ± 8.33	< 0.001
IL-17	31.93 ± 4.59	26.85 ± 3.21	< 0.001
basic FGF	68.80 ± 13.50	56.72 ± 7.55	< 0.001
VEGF	345.94 ± 224.68	195.14 ± 117.56	0.004
IL-15	32.96 ± 4.43	30.62 ± 2.48	0.051
GM-CSF	20.80 ± 14.68	20.55 ± 7.49	0.394

Levels are expressed as the mean ± SD pg/ml. *basic FGF* basic fibroblast growth factor; *GM-CSF* granulocyte-macrophage colony-stimulating factor, *ICNV* idiopathic choroidal neovascularization, *IL* interleukin, *VEGF* vascular endothelial growth factor

### Correlations between serum cytokines and clinical parameters

We aimed to identify potential cytokine markers associated with clinical parameters including BCVA, CRT and lesion area at baseline. Interestingly, as shown in Table 2, the GM-CSF levels in serum were positively related to CRT (*r* = 0.519, *P* = 0.002), and the IL-17 levels in serum were positively related to lesion area (*r* = 0.735, *P* = 0.004). The serum levels of IL-2, IL-10, IL-15, basic FGF, and VEGF did not correlate significantly with BCVA, CRT, or lesion area.

**Table 2:** Correlations between the serum cytokines and BCVA, CRT, or lesion area

	BCVA		CRT		Lesion Area	
Variables	*r*	*P* Value	*r*	*P* Value	*r*	*P* Value
IL-2	−0.061	0.745	0.203	0.266	0.276	0.361
IL-10	−0.312	0.093	0.175	0.347	0.121	0.694
IL-15	0.134	0.472	0.089	0.627	0.478	0.099
IL-17	−0.095	0.610	−0.021	0.911	0.735	0.004
basic FGF	−0.125	0.503	0.072	0.697	0.382	0.197
GM-CSF	0.023	0.901	0.519	0.002	0.172	0.573
VEGF	0.171	0.359	0.190	0.297	−0.228	0.456

Correlation coefficient (*r*) and *P* values are calculated by Spearman’s correlation. *Basic FGF* basic fibroblast growth factor, *BCVA* best corrected visual acuity, *CRT* central retinal thickness, *GM-CSF* granulocyte-macrophage colony-stimulating factor, *IL* interleukin, *VEGF* vascular endothelial growth factor

## Discussion

Recent studies have shown that systemic inflammation is highly likely to be involved in the development and progression of AMD [19]. Higher systemic levels of inflammatory cytokines including IL-1α, IL-1β, IL-2, IL-4, IL-5, IL-10, IL-13, IL-17, TNF-α, soluble TNF receptor II, and VEGF were found to be associated with AMD compared to controls [16, 20–22]. However, only a limited number of systemic cytokines have been investigated in patients with ICNV [23]. In the present study, we analysed the systemic cytokine profile of patients with ICNV and control subjects by means of a multiplex sampling approach, and we demonstrated that patients with ICNV have elevated systemic levels of the inflammatory cytokines IL-2, IL-10, IL-17, basic FGF, and VEGF.

IL-17, mainly secreted by T-helper 17 cells, is an early promoter of inflammatory response that may contribute to tumor angiogenesis by inducing the expression of angiogenic factors, including VEGF, prostaglandin E2 [24]. IL-17 is also secreted by γδ T-cells and innate lymphoid cells [25]. Recently, it has been demonstrated that IL-17 has a strong potential for stimulating neovascularization in a VEGF-independent manner in a laser-induced mouse CNV model [25]. Furthermore, serum and intraocular levels of IL-17 have been shown to be elevated significantly in patients with neovascular AMD, suggesting that IL-17 may be involved in the pathogenesis of neovascular AMD by promoting CNV [20, 26]. In addition to IL-17, IL-2, IL-10, basic FGF, and VEGF appear to be involved in the pathogenesis of CNV [27–31].

We also attempted to determine whether increased serum inflammatory cytokines in ICNV correlate with disease severity. We found that serum IL-17 levels significantly correlated with lesion area. Interestingly, we also found serum GM-CSF levels significantly correlated with CRT. GM-CSF promotes the survival and activation of macrophages and microglia [32]. A study from Wang et al. found that GM-CSF is elevated in the vitreous humour of eyes having the at-risk homozygous CC variant in the complement factor H (CFH) gene [33]. The polymorphism in the CFH gene is a known genetic risk factor for AMD; this is supported by the notion that the CFH Y402H single nucleotide polymorphism is associated with a state of inflammation characterized by increased proinflammatory molecules systemically in circulation system and locally in eye tissue [33]. Thus, as in cases of AMD, GM-CSF may have a relevant role in the inflammatory phenomenon seen in ICNV.

With access to the outer retina via the choroidal vasculature, the inflammatory cytokines that increased in our study may predispose the local retinal environment to a proinflammatory status and promote the development of CNV. We speculate that, like neovascular AMD, ICNV might be a disease with a systemic low-level inflammatory environment, which is the organ-specific manifestation of the subclinical systemic inflammation, as suggested by evidence of similar elevated inflammatory cytokines in serum and aqueous humour [17]. In agreement with this hypothesis, Giovannini et al. reported that the use of systemic steroid before PDT has shown better BCVA outcome than PDT alone for the management of ICNV [34]. Moreover, PDT preceded by systemic steroid could reduce the mean number of PDT applications and produce smaller scars and less hyperplastic fibrosis [34]. Considering that anti-VEGF therapy was superior to PDT for ICNV [35], we hypothesize that combined anti-VEGF and anti-inflammatory treatment might be a better strategy for the treatment of ICNV. Theoretically, the active process of neovascularization in the choroid may also be the source of the significantly increased serum inflammatory cytokine levels in patients with ICNV. However, since the rather small lesion size of CNV does not seem to affect serum cytokine levels, it seems more likely that elevated serum inflammatory cytokines in patients with ICNV are produced by cells located extraocularly.

The present study has several limitations. First, no follow-up was conducted to investigate serum inflammatory cytokine levels when CNV regressed after ranibizumab treatment. Second, we evaluated the cytokines that were significantly elevated in the aqueous humour of eyes with ICNV; however, we were not able to find changes in concentrations of other important cytokines to reveal their role in the development of ICNV. To study ICNV pathogenesis in detail, further studies with a broader cytokine spectrum are needed.

## Conclusions

Our study shows that ICNV may be associated with high levels of serum IL-2, IL-10, IL-17, basic FGF, and VEGF as compared to healthy controls. Additionally, we showed the association of IL-17 with CNV lesion. This underscores a systemic inflammatory contribution to the disease and may provide potential treatment targets for ICNV.

## Abbreviations

AMD: Age-related macular degeneration
basic FGF: basic fibroblast growth factor
BCVA: Best corrected visual acuity
CFH: Complement factor H
CNV: Choroidal neovascularization
CRT: Central retinal thickness
FFA: Fundus fluorescein angiography
GM-CSF: Granulocyte-macrophage colony-stimulating factor
ICNV: Idiopathic choroidal neovascularization
IFN-γ: Interferon (IFN)-γ
IL: Interleukin
logMAR: logarithm of the minimum angle of resolution
OCT: Optic coherence tomography
PDT: Photodynamic therapy
PM: Pathologic myopia
SD: Standard deviation
TNF-α: Tumor necrosis factor-α
VEGF: Vascular endothelial growth factor
